# Serum metabolic signatures of nutritional status in early childhood: results from the Brazilian National Survey on Child Nutrition (ENANI-2019)

**DOI:** 10.1007/s00394-026-04101-9

**Published:** 2026-09-12

**Authors:** Samary da Silva Rosa Freire, Victor Nahuel Keller, Paula Normando, Raquel Machado Schincaglia, Nathalia Cristina Freitas-Costa, Inês Rugani Ribeiro de Castro, Zachary Kroezen, Meera Shanmuganathan, Philip Britz-McKibbin, Gilberto Kac

**Affiliations:** 1 Nutritional Epidemiology Observatory, Josué de Castro Nutrition Institute, Federal University of Rio de Janeiro, Rio de Janeiro, Brazil; 2 Nutrition and Dietetics, Josué de Castro Nutrition Institute, Federal University of Rio de Janeiro, Rio de Janeiro, RJ Brazil; 3 Nutrition Faculty, Federal University of Goiás, Goiânia, GO Brazil; 4 Institute of Nutrition, State University of Rio de Janeiro, Rio de Janeiro, Brazil; 5 Department of Chemistry and Chemical Biology, McMaster University, Hamilton, Canada; 6 Cidade Universitária, Avenida Carlos Chagas Filho, 373, J2, room 29, Rio de Janeiro, RJ 21941902 Brazil

**Keywords:** Metabolomics, Infant nutrition, Stunting, Overweight, Sex-specificity

## Abstract

**Background:**

Untargeted metabolic profiling may provide biochemical insights into adverse growth outcomes in early childhood. We aimed to investigate the association between serum metabolic signatures and anthropometric indicators in young children.

**Methods:**

The serum metabolome of 4978 children aged 6–59 months, studied by the 2019 Brazilian National Survey on Child Nutrition (ENANI-2019), was analyzed using multi-segment injection-capillary electrophoresis-mass spectrometry. WHO length/height-for-age (HAZ), weight-for-height (WHZ), body-mass-index-for-age (BMIZ), and weight-for-age (WAZ) indices, and stunting (HAZ <-2) and overweight (WHZ > 2) were the outcomes. Partial least squares regression was used to select serum metabolites with a variable importance projection ≥ 1. Linear and logistic regression models, adjusted for confounders and sex-stratified, were fitted. The Benjamini–Hochberg correction (adjusted *p* < 0.05) controlled the false discovery rate.

**Results:**

Tyrosine, creatinine, and 5-aminovaleric acid were consistently directly associated with all anthropometric indices. Threonine was directly associated with HAZ. The branched-chain amino acids were directly associated with WHZ. Leucine, valine, lysine, and carnitine were directly associated with BMIZ, whereas 3-hydroxybutyric acid was inversely associated with WAZ. Direct associations between methionine and WHZ, BMIZ, and WAZ, and between histidine and WHZ and BMIZ, were observed exclusively in girls, while hippuric acid and cresol sulfate were inversely associated with HAZ, and phenylacetylglutamine with WAZ, exclusively in boys. Further, 2-aminobutyric acid and creatinine were inversely associated with stunting, and proline betaine was inversely associated with being overweight.

**Conclusion:**

Serum metabolomics offers a valuable tool for screening for childhood malnutrition, and key metabolites were associated with energy and muscle metabolism, the endocrine system, and aspects of the human diet.

**Supplementary Information:**

The online version contains supplementary material available at https://doi.org/10.1007/s00394-026-04101-9.

## Background

Early childhood is considered a critical period for health and nurturing care, playing a decisive role in the child’s life course [1, 2]. Adequate nutrition is a key component of nurturing care and supports normal child growth [3]. Therefore, in line with the World Health Organization (WHO) recommendations, children should have their anthropometric measurements taken regularly to detect all forms of malnutrition [4].

Despite WHO guidelines, the 2025 Joint Child Malnutrition Estimates indicated insufficient progress toward the nutrition targets: approximately 150 million children under five are stunted, 42.8 million are wasted, and 35.5 million are overweight. This indicates a growing shift in the burden of childhood nutritional diseases from undernutrition to overnutrition, reflecting excess caloric intake in low- and middle-income countries [5]. This shift could be attributed to obesogenic environments, often characterized by food deserts and food swamps, alongside persistent socioeconomic inequalities [6, 7].

All forms of child malnutrition can have long-term consequences throughout the life course, rendering older children and adults more susceptible to cardiometabolic disorders, infectious diseases, weakened immunity, learning difficulties, and developmental delays [5]. Metabolomic studies are considered a valuable tool for better understanding physiological and metabolic adaptations to malnutrition in early childhood [8]. Recent advances in metabolomics can provide mechanistic insights into childhood malnutrition by measuring metabolic responses to exposomics at critical stages of development [9]. This includes differentiating the metabolic phenotype of malnourished children across stages of severity, as well as of surviving adults who suffered from malnutrition in infancy [5, 10].

Examples of metabolomic studies of metabolome associated with child malnutrition are relatively scarce. Azab et al. [11] in a cross-sectional analysis of two Canadian birth cohorts of 900 Canadian children aged 5 years, found a direct association between circulating concentrations of branched-chain and aromatic amino acids and overweight and an excess adiposity phenotype, with the association more prominent in female than in male children [11]. Moreau et al. [12], with 130 children in Bangladesh, found a positive association between plasma threonine at 9 and 36 months and the height/length-for-age z-score (HAZ) [12].

Given the importance of public health policies that promote early childhood growth and the lack of data on malnutrition biomarkers in Brazilian children, this study aims to explore the association between the serum metabolome and the nutritional status of children aged 6–59 months based on data from the Brazilian National Survey on Child Nutrition (ENANI-2019, from the Portuguese *Estudo Nacional de Alimentação e Nutrição Infantil*)).

## Methods

### Study design and participants

The present study uses data from ENANI-2019, a population-based household survey of children under five years old (< 5y). The ENANI-2019 investigated breastfeeding practices, dietary intake, anthropometric status, micronutrient deficiencies, and the serum metabolome. Data were collected between February 2019 and March 2020, and the study design details were previously described [13, 14].

The following conditions were not considered eligible for this study: households in which indigenous people lived in villages; foreigners who did not speak Portuguese; children with any condition that prevented them from having their anthropometric measurements taken; and residents of collective residences (hotels, guesthouses and similar establishments, orphanages, and hospitals).

### Blood collection

A trained professional collected the blood sample during a scheduled household visit. It was not necessary to fast or avoid usual medications. Samples were centrifuged to obtain serum, which was transferred to light-protected tubes and stored at − 20 °C. These were identified by each child’s barcode and stored at -80 °C in a biorepository. Further details of the sample collection protocol were described elsewhere [13].

### Serum processing and metabolome analysis

Untargeted serum metabolomic analyses were conducted at the Department of Chemistry and Chemical Biology at McMaster University (Hamilton, Canada). The 5004 serum samples archived at the biorepository were shipped frozen by courier at a controlled temperature. Padilha et al. [15] previously described the standardized protocol and data workflow for serum metabolomic analyses. Briefly, a comprehensive analysis of polar/ionic metabolites from diluted serum filtrate samples was carried out using multisegment injection-capillary electrophoresis-mass spectrometry (MSI-CE-MS) with an Agilent 6230B time-of-flight mass spectrometer (Agilent, Santa Clara, USA) with a coaxial sheath flow electrospray interface coupled to an Agilent G7100A capillary electrophoresis (CE) instrument (Agilent, Santa Clara, USA).

Data quality was evaluated by repeated analysis of a pooled quality control (QC) and internal standards added to all serum samples using a multiplexed separation format comprising 13 independent samples analyzed in a single analytical run [16]. Serum filtrate samples were analyzed by MSI-CE-MS in two configurations under positive (cationic metabolites) and negative (anionic metabolites) ionization modes across three batches of 979, 1990, and 2035 serum samples from ENANI-2019 [17, 18]. Overall, 73 polar metabolites in serum filtrate samples were measured with adequate technical precision, with a mean coefficient of variation (CV) < 30% for pooled QC samples. We applied a robust QC-based batch correction algorithm [19] to serum metabolomics data from ENANI-2019 to correct for long-term signal drift, leveraging the serial-injection format of MSI-CE-MS, in which each analytical run includes a randomly positioned QC [20]. Known serum metabolites were reported as concentrations (µmol/L) using external calibration curves. In contrast, unknown metabolites were assessed based on their ion responses, measured as relative peak areas (RPA), and annotated by their characteristic accurate mass (*m/z*), relative migration time (RMT), and ionization mode (p or n) in MSI-CE-MS [21].

Lastly, we analyzed missing values according to Wei et al. (2018) recommendations, which exclude metabolites with more than 20% missing values; however, we adopted a less rigorous strategy, using a threshold of 50% rather than 20%. Based on Wei et al. (2018) recommendation, all 73 serum metabolites satisfied the above selection criteria and were not excluded; thus, the missing data were imputed using the random forest (RF) method, and non-detection missing data were evaluated using quantile regression imputation of left-censored data (QRILC) [21].

### Anthropometric measurements

Weight (kg) and height/length (cm) were obtained by trained personnel, with children wearing light clothing and barefoot, under standardized conditions.

The weight of children < 2 years was measured using a digital pediatric scale (model 336; SECA, Hamburg, Germany). For children ≥ 2 years, the platform scale (model 803; SECA, Hamburg, Germany) was used. The length of children < 2 years of age was measured with the child lying down on an infantometer (model 417; SECA, Hamburg, Germany), and the height of children ≥ 2 years was measured while standing on a stadiometer (model 213; SECA, Hamburg, Germany). All measurements were performed in duplicate in accordance with the study protocol, and the equipment was calibrated before the procedures. More details on the methods are described by dos Anjos et al. [22].

The anthropometric data were evaluated using the quality indicator, as recommended by WHO/UNICEF. Implausible measures were classified according to WHZ < -5 or > 5, HAZ <-6 or > 6, and weight-for-age (WAZ) <-6 or > 5 [4]. We handled missing or implausible values using the “nearest neighbor” imputation method, in which socio-economic and demographic information and existing anthropometric measurements were considered when selecting donor records [23].

HAZ, WHZ, BMIZ, and WAZ were calculated and classified according to the WHO-recommended Child Growth Standards, using the Anthro package (https://cran.r-project.org/web/packages/anthro/index.html) [24]. Malnutrition was classified as stunting (HAZ < -2), wasting (WHZ < -2), underweight (WAZ < − 2), and overweight (WHZ > 2).

### Covariates

A structured questionnaire was administered to the child’s caregiver to collect data on: child sex (male/female), date of birth, household region (Midwest/Northeast/North/Southeast/South), birth weight (g) and length (cm), gestational age at delivery (weeks), and exclusive breastfeeding practices according to WHO [25]. The per-capita family income was categorized according to the Brazilian minimum wage (R$998.00) and converted to the United States dollar exchange rate (R$4.013 = USD 1), as of December 30, 2019, into four groups (0–62.2, 62.3–124.4, 124.5–248.7, or > 248.7 USD). Food insecurity was categorized as severe, mild, moderate, or food security according to the Brazilian Household Food Insecurity Measurement Scale (EBIA), which comprises 14 items measuring household food access over the past three months [26–28].

Early childhood development (ECD) was assessed using the Survey of Well-being of Young Children (SWYC) milestones questionnaire, and the developmental quotient (DQ) was calculated by dividing developmental age by chronological age. The developmental age was determined based on specific developmental milestones achieved, estimated using item response theory and graded response models [15, 29, 30]. This methodology was indicated by the instrument’s creators and described in detail previously [29, 30].

The child’s diet quality was assessed using the minimum dietary diversity (MDD) indicator proposed by WHO [25]. To meet the MDD criteria, a child must have consumed at least five of the following eight food groups on the previous day of the visit: [1] breast milk; [2] grains, roots, tubers, and plantains; [3] pulses (beans, peas, lentils), nuts, and seeds; [4] dairy products (milk, infant formula, yogurt, cheese); [5] flesh foods (meat, fish, poultry, organ meats); [6] eggs; [7] vitamin-A rich fruits and vegetables; and [8] other fruits and vegetables. The variable was dichotomized into < 5 and ≥ 5 food groups. These data were obtained using a 24-hour recall from the structured ENANI questionnaire [31].

### Statistical analysis

The descriptive analysis used measures of central tendency for continuous variables and absolute (n) and relative (%) frequencies for categorical variables. 95% confidence intervals were also estimated.

Pearson correlation coefficients were calculated to assess associations between metabolites and HAZ, WHZ, BMIZ, and WAZ. A heatmap graph was created to visualize these results using the ComplexHeatmap package (https://jokergoo.github.io/ComplexHeatmap-reference/book/).

A Partial Least Squares Regression (PLS-R) model was fitted to select discriminating serum metabolites and classify their contributions by outcome and avoid multicollinearity. We used variable importance projection (VIP) for this purpose [32]. The PLS-R employed 80% of the data for training and 20% for testing, with random selection. The model with the optimal number of components was chosen to generate VIP scores ≥ 1. This analysis utilized the following packages: pls (Mevik and Wehrens, 2007) and plsVarSel (https://github.com/khliland/plsVarSel).

A Directed Acyclic Graph (DAG), a diagram of causal relationships between exposure and outcomes, was defined to perform minimal adjustments based on the identified confounders, using the DAGitty software, freely available at https://dagitty.net/ [33]. The DAG identified the following variables as potential confounders that were adjusted for in the regression models: birth weight, breastfeeding, child’s age, child development, per-capita family income, food diversity, food insecurity, and preterm birth (Supplementary Fig. 1). The exclusive breastfeeding indicator was collected only for children aged 0–23 months. Therefore, it wasn’t a candidate for adjustment analysis. Birth weight was retained as a confounder, as indicated by the DAG; implausible values falling outside the INTERGROWTH-21st thresholds (z-score ≤ − 4 or ≥ + 5) were excluded, corresponding to 0.5% of the observations. These confounders were not included in the adjustment. The other variables had no missing values.

To explore how macroregion and family income relate to child malnutrition, a Pearson chi-square test was performed to examine the association between stunting and overweight categories and per capita family income and macroregion. To identify differences, cell-wise adjusted residuals were computed and interpreted using the adjusted alpha [34].

The panel of circulating metabolites selected by PLS-R was included in simple linear and logistic regression models, adjusted for the confounders identified in the DAG, to estimate the association between the serum metabolome and the child’s nutritional status. The linear models used the nutritional indices HAZ, WHZ, BMIZ, and WAZ as outcomes. The logistic model was built using HAZ’s selected metabolites to fit the stunting model, WHZ’s selected metabolites to fit the models of association with wasting and being overweight separately, and WAZ’s selected metabolites to fit the underweight model. In a dataset of 4978 observations, the outcome variables WHZ, wasting, and overweight had 24 missing values; BMIZ had 8; and HAZ, WAZ, underweight, and stunting had 1 missing value each. The missing values were excluded from the analysis.

The regression models were also stratified by sex to test the hypothesis of sex dependence for certain metabolites [11]. Also, a linear model was built to observe the association between the metabolome and child sex (Supplementary Table 1). Statistically significant results were considered with p-values < 0.05 after a Benjamini–Hochberg correction for multiple comparisons to control the false discovery rate. All serum metabolites were expressed in standard deviation units to permit direct comparison. The analytical workflow is illustrated to clarify the decision-making process (Supplementary Fig. 2).

For data management, the dplyr package and its dependencies (tidyverse, tibble, and tidyr) were used; for figure generation, ggplot2 and its dependencies (cowplot, ggrepel), circlize, grid, gridExtra, gridGraphics, and reshape2 were used. A Venn diagram was also built to show overlap among the serum metabolites, using the packages VennDiagram and gVennDiagram. Statistical analyses were carried out using the R programming language (R Core Team; http://www.R-project.org) in JupyterLab.

A pathway analysis was conducted for serum metabolites identified through linear and logistic regression analyses to identify underlying metabolic pathways. A metabolic network was used in Cytoscape [35] using the MetScape plugin with the KEGG database.

## Results

### Descriptive participant characteristics

**Fig. 1:**
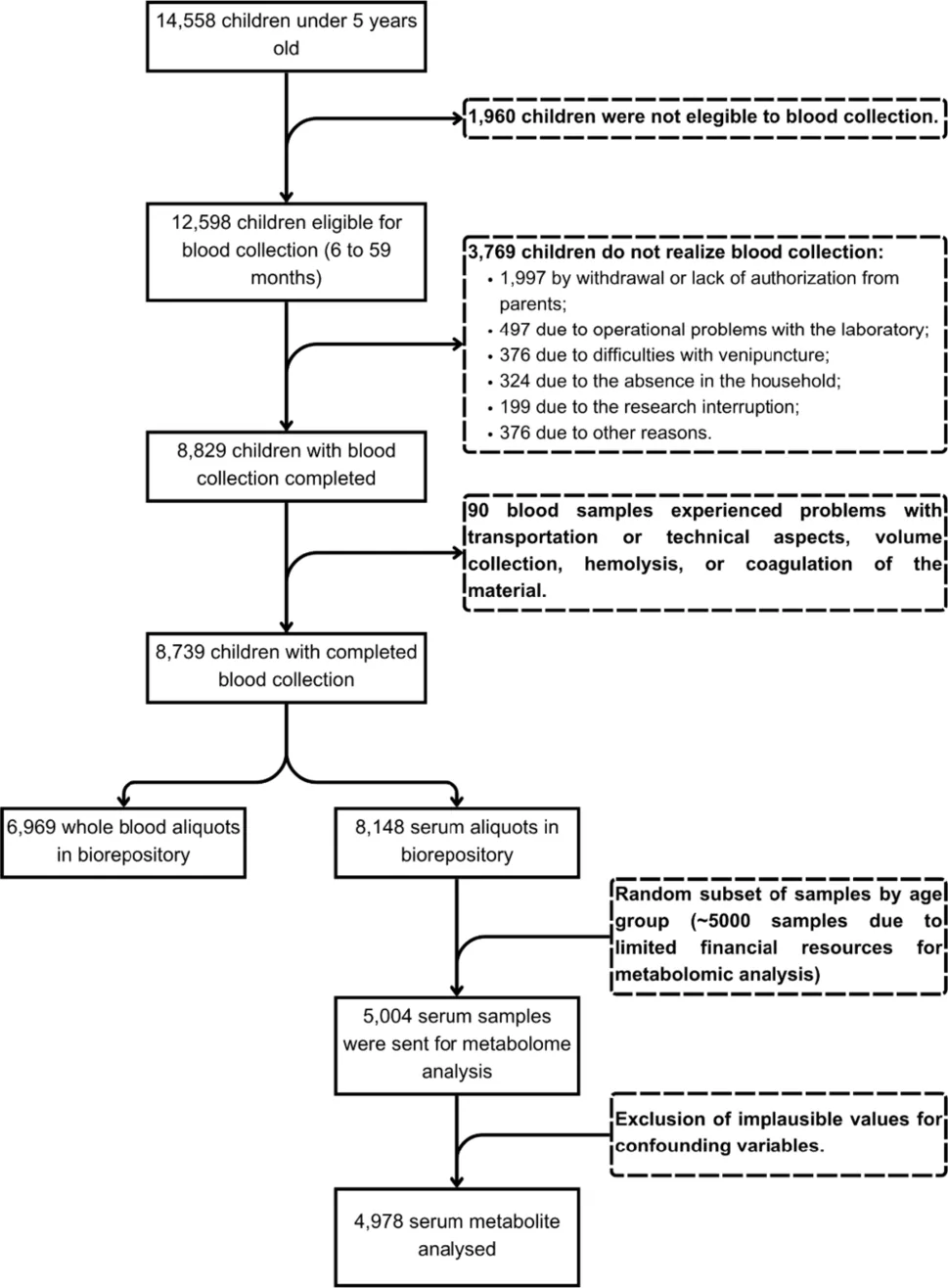
Flowchart of data collection of ENANI-2019

The ENANI-2019 study enrolled 14,558 children aged 0–59 months living in 12,524 households; among them, 12,598 children aged 6–59 months were considered eligible for drawing blood samples. However, only 8829 children had blood specimens collected, and 5004 serum samples were selected using a random sampling procedure to ensure representation across the original sample distribution; of these, 4978 were analyzed (Fig. 1).

The prevalence of stunting, wasting, and overweight was 5.7%, 1.6%, and 7.3%, respectively, according to WHO standards. Additionally, 12.0% of children were born small for gestational age, 18.1% large for gestational age, and 12.2% were preterm (Table 1).

**Table 1:** Descriptive characteristics of children aged 6 to 59 months assessed in a subset sample of ENANI-2019

Variables		Mean or frequency (%)	95% CI
*Region*			
	Midwest	20.5	19.4; 21.6
	Northeast	22.5	21.3; 23.7
	North	24.8	23.6; 26
	Southeast	18.5	17.4; 19.6
	South	13.5	12.6; 14.4
*Age group (months)*			
	6–23	29.6	28.3; 30.9
	24–35	21.5	20.4; 22.6
	36–59	48.9	47.5; 50.3
*Sex*			
	Male	51.1	49.7; 52.5
	Female	48.9	47.5; 50.3
*Preterm birth*			
	No	87.8	86.9; 88.7
	Yes	12.2	11.3; 13.1
*Per-capita family income (USD)* ^1^			
	< 62.20	31.4	30.1; 32.7
	62.20-124.40	33.9	32.6; 35.2
	124.50-248.70	24.5	23.3; 25.7
	> 248.70	10.1	9.3; 10.9
*Food security* ^2^			
	Severe insecurity	5.7	5.1; 6.3
	Mild insecurity	39.0	37.6; 40.4
	Moderate insecurity	8.4	7.6; 9.2
	Food security	46.9	45.5; 48.3
*Minimum dietary diversity (MDD)* ^3^			
	< 5 food groups	40.7	39.3; 42.1
	≥ 5 food groups	59.3	57.9; 60.7
*Stunting* ^4^			
	No	94.3	93.7; 94.9
	Yes	5.7	5.1; 6.3
*Wasting* ^5^			
	No	98.4	98.1; 98.7
	Yes	1.6	1.3; 1.9
*Overweight* ^6^			
	No	92.7	92.0; 93.4
	Yes	7.3	6.6; 8
*Underweight* ^7^			
	No	97.9	97.5; 98.3
	Yes	2.1	1.7; 2.5
*Birth weight for gestational age* ^8^			
	Small for Gestational Age (< P10)	12.0	11.1; 12.9
	Adequate for Gestational Age (P10-P90)	70	68.7; 71.3
	Large for Gestational Age (> P90)	18.1	17.0; 19.2
Length/height for age (z-score) ^9^		-0.3	-0.3; -0.3
Weight for Length/height (z-score) ^9^		0.3	0.3; 0.3
Body mass index for age (z-score) ^9^		0.4	0.4; 0.4
Weight for age (z-score) ^9^		0.1	0.1; 0.1
Child developmental quotient (DQ) ^10^		0.98	0.97;0.99

^1^Estimated according to the Brazilian minimum wage (R$998.00) and converted to the USD exchange (R$4.013 = USD 1) on December 31st, 2019, where the minimum Brazilian salary was USD 248.70. ^2^Food security was measured according to the Brazilian Food Insecurity Scale (EBIA – *Escala Brasileira de Insegurança Alimentar*), which consists of 14 items. ^3^MDD: is characterized by the frequency with which children received ≥ 5 or < 5 of the eight food groups the day before the interview. [food groups: [1] breast milk; [2] grains, roots, and tubers; [3] beans, nuts, and seeds; [4] dairy products; [5] flesh foods; [6] eggs; [7] vitamin A-rich fruits and vegetables; and [8] fruits and vegetables].^4^Stunting (length/height-for-age z-score < -2), ^5^Wasting (weight-for-length/height z-score < -2), ^6^Overweight (weight-for-length/height z-score > 2), ^7^Underweight (weight-for-age z-score < -2). ^8^The birth weight for gestational age was calculated according to sex and gestational age-specific percentiles using the INTERGROWTH-21st charts.^9^ The z-score of anthropometric measurements was calculated according to the World Health Organization (WHO) child growth standards. ^10^Developmental quotient (DQ) was calculated using the Survey of Well-being of Young Children’s - Brazilian version (SWYC-BR) milestones questionnaire. CI: confidence interval; USD: United States Dollar

The Pearson chi-square test showed a significant association between macro-region and stunting (*p* = 0.01), macro-region and overweight (*p* = 0.0001), and per-capita family income and stunting (*p* = 0.04); however, after exploring the adjusted residuals, we observed that the prevalence of stunting according to macro-region in the North region was significantly higher (*p* = 0.002), and the prevalence of overweight was higher than expected in the South region (*p* = 0.0001), after the adjustment non-significant differences were found with per-capita family income (Supplementary Table 2).

### Correlation between serum metabolites and anthropometric z-scores and metabolite selection by PLS-R

**Fig. 2:**
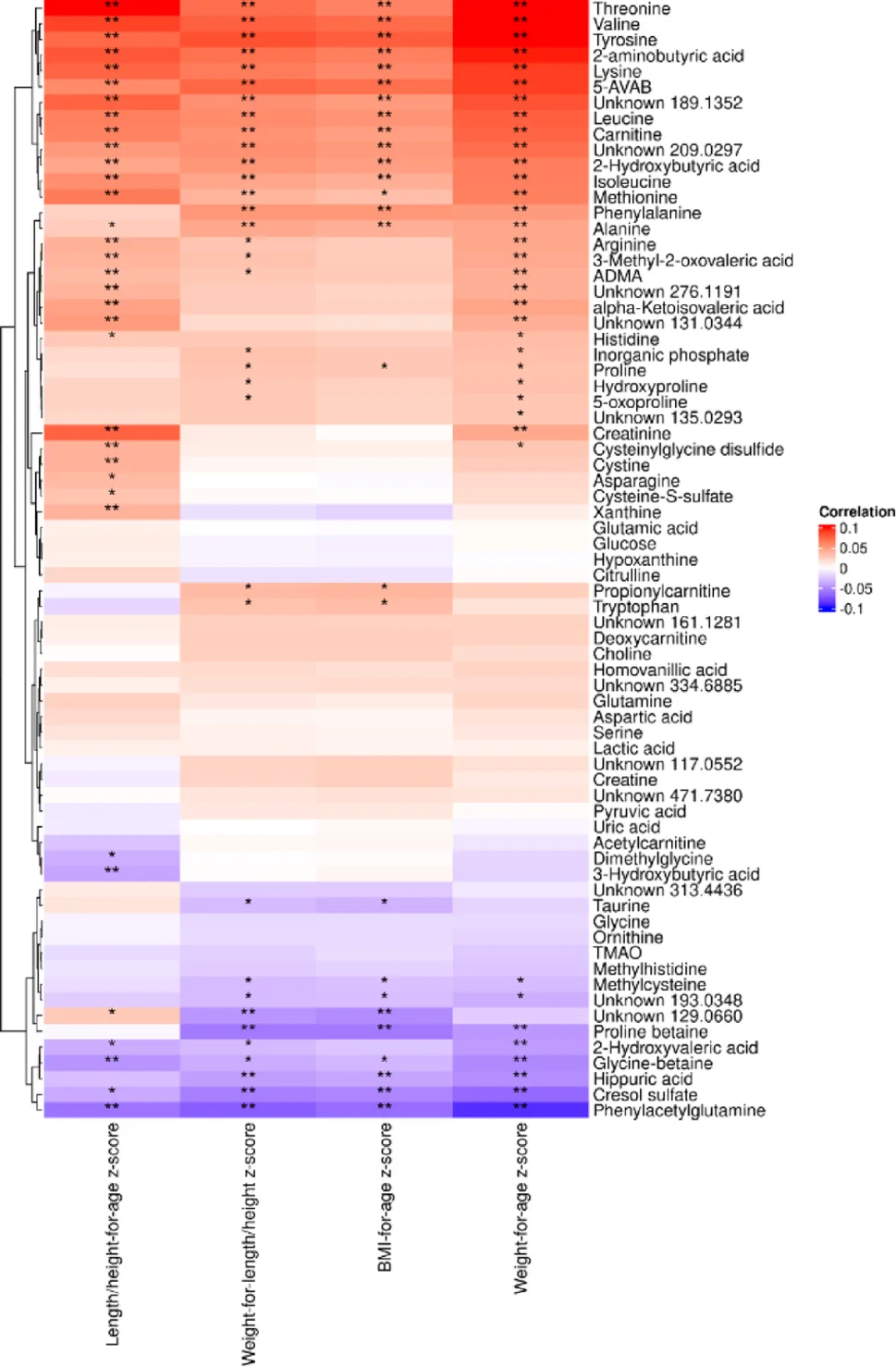
Correlation heatmap between all serum metabolites and z-score child growth. *Note*: Pearson correlation between all metabolites in the sample and anthropometric indices according to the World Health Organization standard of child growth. Shades of red represent positive correlation, shades of blue negative correlation, and white no correlation. * *p*-value < 0.05; ** *p*-value < 0.01

Pearson correlation analysis revealed that threonine, valine, tyrosine, 2-aminobutyric acid, lysine, 5-AVAB, leucine, carnitine, 2-hydroxybutyric acid, isoleucine, and methionine were directly correlated with all anthropometric indices. In contrast, glycine betaine, cresol sulfate, and phenylacetylglutamine were inversely correlated with all the anthropometric indices. Nonetheless, the metabolites phenylalanine and proline, which were directly correlated with WHZ, BMIZ, and WAZ, did not show an association with HAZ, whereas proline betaine was inversely correlated with WHZ, BMIZ, and WAZ. Acetylcarnitine and Dimethylglycine showed only an inverse correlation with HAZ. (Fig. 2).

**Fig. 3:**
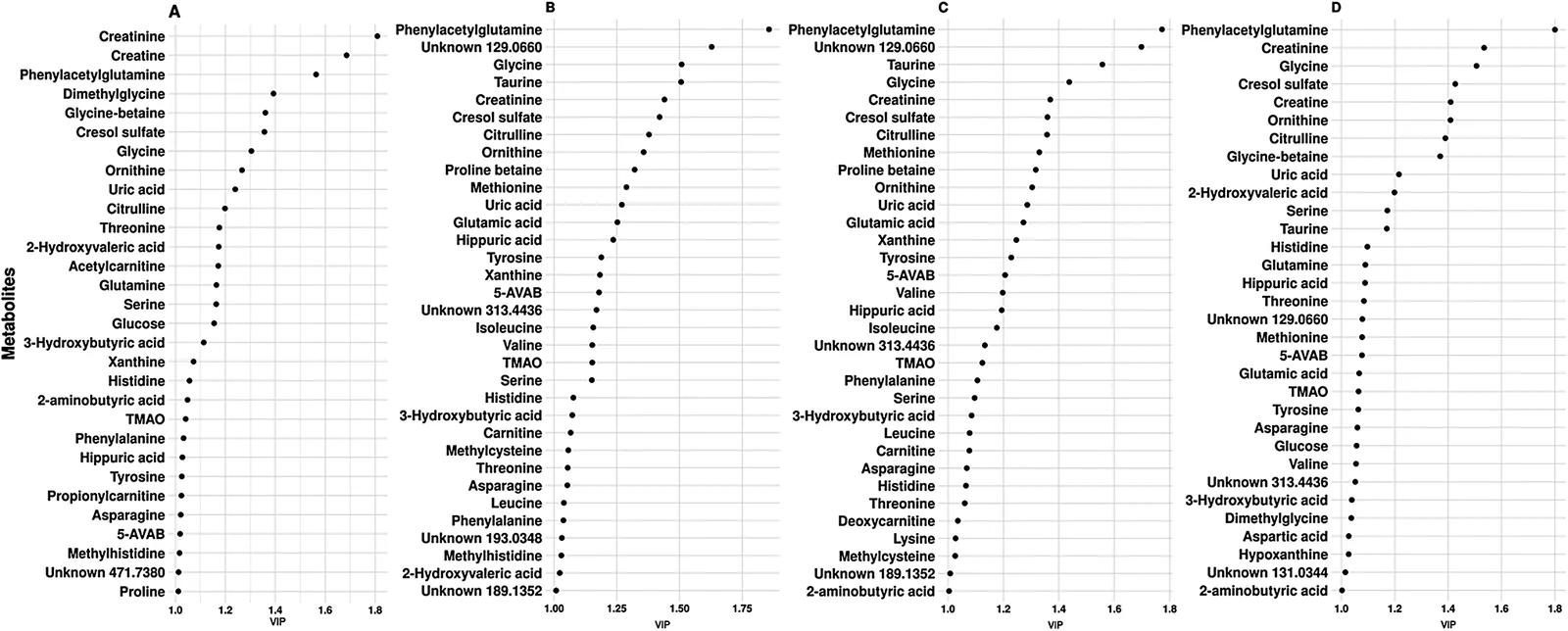
Variable importance projection (VIP) ranking scores of metabolites from Partial Least Squares Regression (PLSR) analysis for the z-score child growth standard. *Notes*: The most relevant metabolites ranked by the PLSR that explain the following outcomes: **A**: Length/height-for-age z-score. **B**: Weight-for-length/height z-score. **C**: Body mass index (BMI)-for-age z-score. **D**: Weight-for-age z-score. The z-scores for anthropometric measurements were calculated according to the World Health Organization (WHO) child growth standards. Only metabolites with VIP scores ≥ 1 are displayed. Higher VIP scores indicate major contributions, while lower VIP scores indicate minor contributions in explaining z-score variability in child growth. 5-AVAB: 5-aminovaleric-acid-betaine. TMAO = trimethylamine-N-oxide

A total of 73 serum metabolites were included as variables in a PLS-R model having HAZ, WHZ, BMIZ, and WAZ as outcomes. Specifically, HAZ, WHZ, BMIZ, and WAZ identified 31, 34, 32, and 32 metabolites, respectively; none of these metabolites did not overlap completely (Fig. 3).

The serum metabolites creatinine, phenylacetylglutamine, cresol sulfate, glycine, ornithine, uric acid, citrulline, threonine, serine, 3-hydroxybutyric acid, tyrosine, trimethylamine-*N*-oxide (TMAO), glutamic acid, and the 5-aminovaleric acid betaine (5-AVAB or δ-valerobetaine) (Supplementary Fig. 3) were consistently ranked as important (VIP ≥ 1) across these anthropometric outcomes (Fig. 3, Supplementary Figs. 4, 5, 6 and 7).

### Association of serum metabolomic profiles and anthropometric z-score of metabolites

**Fig. 4:**
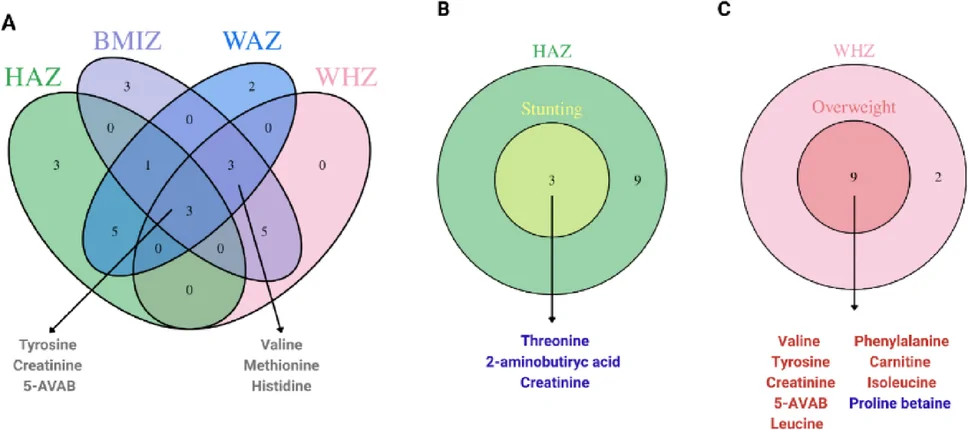
Venn diagram of the shared and not shared metabolites associated with child growth. *Notes*: **A**: The diagram shows the distribution of the metabolites associated with the z-scores of child growth, and the metabolites described in gray are those that are shared between all the classifications and the classifications of weight. **B**: The metabolites were described in blue (decreased in stunting). **C**: The metabolites were described in red (increased in overweight) and blue (decreased in overweight). HAZ: length/height-for-age z-score. WHZ: weight-for-length/height z-score. BMIZ: body-mass-index-for-age z-score. WAZ: weight-for-age z-score

It was observed that the serum metabolites tyrosine, creatinine, and 5-AVAB were directly associated with all anthropometric indices, and valine, methionine, and histidine were directly associated with the three weight anthropometric indices (WHZ, BMIZ, and WAZ) (Fig. 4A).

Direct associations were observed between serum threonine (β = 0.11), 2-aminobutyric (β = 0.09), creatinine (β = 0.08), tyrosine (β = 0.08), 5-AVAB (β = 0.07), and xanthine (β = 0.05), alanine (β = 0.04) and the HAZ, whereas phenylacetylglutamine (β = − 0.08), glycine betaine (β = − 0.05), cresol sulfate (β = − 0.05), 2-hydroxyvaleric acid (β = − 0.04) and hippuric acid (β = − 0.04) were inversely associated (Table 2). Also, serum valine (β = 0.10), tyrosine (β = 0.10), an unknown cation (189.1352 *m/z*; C_10_H_20_OS) (β = 0.09), 5-AVAB (β = 0.09), creatinine (β = 0.09), leucine (β = 0.08), phenylalanine (β = 0.08), carnitine (β = 0.07), methionine (β = 0.06), isoleucine (β = 0.06) and histidine (β = 0.05) showed direct associations with WHZ. In contrast, serum proline betaine (β = − 0.06) exhibited an inverse association (Table 2).

**Table 2:** Association between serum metabolites and anthropometric z-score for children 6–59 months evaluated in a subset sample of ENANI-2019

Metabolites^1^	β^2^	95% CI^3^	*p*-values^4^
*Height-for-age z-score* ^5^			
Threonine	0.11	0.08;0.14	4 × 10^−11^
2-aminobutyric acid	0.09	0.06;0.12	4 × 10^−7^
Tyrosine	0.08	0.05;0.12	9 × 10^−6^
Creatinine	0.08	0.05;0.12	2 × 10^−6^
Phenylacetylglutamine	− 0.08	− 0.11; − 0.05	2 × 10^−5^
5-AVAB	0.07	0.04;0.10	1 × 10^−4^
Glycine betaine	− 0.05	− 0.08; − 0.02	3 × 10^−3^
Xanthine	0.05	0.01;0.08	0.01
Cresol sulfate	− 0.05	− 0.08;− 0.02	0.01
2-Hydroxyvaleric acid	− 0.04	− 0.08; − 0.02	0.03
Hippuric acid	− 0.04	− 0.07; − 0.01	0.02
Alanine	0.04	0.01;0.07	0.04
*Weight-for-height z-score* ^5^			
Valine	0.10	0.07;0.14	8 × 10^−9^
Tyrosine	0.10	0.07;0.14	8 × 10^−9^
Unknown cation (189.1352 *m/z*; C_10_H_20_OS)	0.09	0.05;012	2 × 10^−6^
5-AVAB	0.09	0.05;0.12	8 × 10^−7^
Creatinine	0.09	0.06;0.13	3 × 10^−6^
Leucine	0.08	0.05;0.11	7 × 10^−6^
Phenylalanine	0.08	0.05;0.11	1 × 10^−5^
Carnitine	0.07	0.04;0.10	2 × 10^−4^
Methionine	0.06	0.03;0.10	5 × 10^−4^
Isoleucine	0.06	0.03;0.10	7 × 10^−4^
Proline betaine	− 0.05	− 0.09; − 0.02	2 × 10^−3^
Histidine	0.05	0.02;0.09	4 × 10^−3^
*Body-mass-index z-score* ^5^			
Valine	0.09	0.06;0.13	2 × 10^−7^
Tyrosine	0.09	0.06;0.13	2 × 10^−7^
Unknown cation (189.1352 *m/z*; C_10_H_20_OS)	0.08	0.05;0.11	1 × 10^−5^
5-AVAB	0.08	0.05;0.12	5 × 10^−6^
Lysine	0.08	0.05;0.11	1 × 10^−5^
Threonine	0.07	0.04;0.11	4 × 10^−5^
Phenylalanine	0.08	0.05;0.11	1 × 10^−5^
Creatinine	0.08	0.04;0.11	5 × 10^−5^
Leucine	0.08	0.04;0.11	3 × 10^−5^
Deoxycarnitine	0.06	0.03;0.10	5 × 10^−4^
Carnitine	0.06	0.03;0.09	10 × 10^−4^
Isoleucine	0.06	0.03;0.09	1 × 10^−3^
Methionine	0.06	0.02;0.09	2 × 10^−3^
Proline betaine	− 0.05	− 0.09; − 0.02	3 × 10^−3^
Histidine	0.05	0.01;0.08	1 × 10^−2^
*Weight-for-age z-score* ^5^			
Valine	0.12	0.09;0.15	3 × 10^−14^
Threonine	0.12	0.09;0.15	1 × 10^−13^
Tyrosine	0.12	0.09;0.15	2 × 10^−13^
2-aminobutyric acid	0.10	0.07;0.13	1 × 10^−10^
Creatinine	0.11	0.07;0.14	2 × 10^−9^
5-AVAB	0.10	0.07;0.13	5 × 10^−10^
Methionine	0.09	0.06;0.12	1 × 10^−7^
Phenylacetylglutamine	− 0.06	− 0.10; − 0.03	2 × 10^−4^
Unknown anion (131.034 *m/z*; C_8_H_6_NO)	0.06	0.03;0.09	5 × 10^−4^
Histidine	0.05	0.02;0.08	2 × 10^−3^
Glycine betaine	− 0.06	− 0.09; − 0.02	1 × 10^−3^
3-Hydroxybutyric acid	− 0.04	− 0.07; − 0.01	0.02
Cresol sulfate	− 0.04	− 0.07; − 0.01	0.04
2-Hydroxyvaleric acid	− 0.04	− 0.07; − 0.01	0.04

The serum metabolites, valine (β = 0.09), tyrosine (β = 0.09), an unknown cation (189.1352 *m/z*; C_10_H_20_OS) (β = 0.08), 5-AVAB (β = 0.08), lysine (β = 0.08), threonine (β = 0.07), phenylalanine (β = 0.08), creatinine (β = 0.08), leucine (β = 0.08), deoxycarnitine (also known as γ-butyrobetaine) (β = 0.06), carnitine (β = 0.06), isoleucine (β = 0.06), methionine (β = 0.06) and histidine (β = 0.05) showed a direct association with the BMIZ. In contrast, proline betaine (β = − 0.05) exhibited an inverse association (Table 2). Valine (β = 0.12), threonine (β = 0.12), tyrosine (β = 0.12), 2-aminobutyric acid (β = 0.10), creatinine (β = 0.11), 5-AVAB (β = 0.10), methionine (β = 0.09), an unknown anion (131.034 *m/z*; C_8_H_6_NO) (β = 0.06) and histidine (β = 0.05) were directly associated with WAZ. Meanwhile, circulating levels of phenylacetylglutamine (β = − 0.06), glycine betaine (β = − 0.06), 3-hydroxybutyric acid (β = − 0.04), cresol sulfate (β = − 0.04), and 2-hydroxyvaleric acid (β = − 0.04) showed an inverse association with WAZ (Table 2).

### Association of serum metabolome profile with stunting, wasting, underweight, and overweight

No significant association was found between serum metabolites and wasting or underweight categories in early childhood, given the low prevalence of these categories in ENANI-2019. Nonetheless, serum creatinine (OR = 0.78), threonine (OR = 0.84) and 2-aminobutyric acid (OR = 0.83) were inversely associated with stunting (Fig. 4B; Table 3), whereas valine (OR = 1.27), tyrosine (OR = 1.25), 5-AVAB (OR = 1.24), and phenylalanine (OR = 1.23), creatinine (OR = 1.19), leucine (OR = 1.18) and isoleucine (OR = 1.16) were directly associated with overweight whereas the proline betaine (OR = 0.86) was inversely associated (Fig. 4C; Table 3).

**Table 3:** Serum metabolites associated with malnutrition categories for children 6–59 months evaluated in a subset sample of ENANI-2019

Metabolites^1^	OR^2^	95% CI^3^	*p*-values^4^
*Stunting* ^5^			
Creatinine	0.78	0.68;0.90	0.02
Threonine	0.84	0.75;0.95	0.04
2-aminobutyric acid	0.83	0.73;0.93	0.04
*Overweight* ^6^			
Valine	1.27	1.14;1.41	6 × 10^−4^
Tyrosine	1.25	1.12;1.39	7 × 10^−4^
5-AVAB	1.24	1.12;1.39	9 × 10^−4^
Phenylalanine	1.23	1.10;1.37	1 × 10^−3^
Creatinine	1.19	1.06;1.33	0.02
Leucine	1.18	1.06;1.31	0.02
Proline betaine	0.86	0.77;0.96	0.04
Isoleucine	1.16	1.04;1.30	0.04

### Investigation of sex-dependent differences in metabolite markers associated with anthropometric measurements

**Fig. 5:**
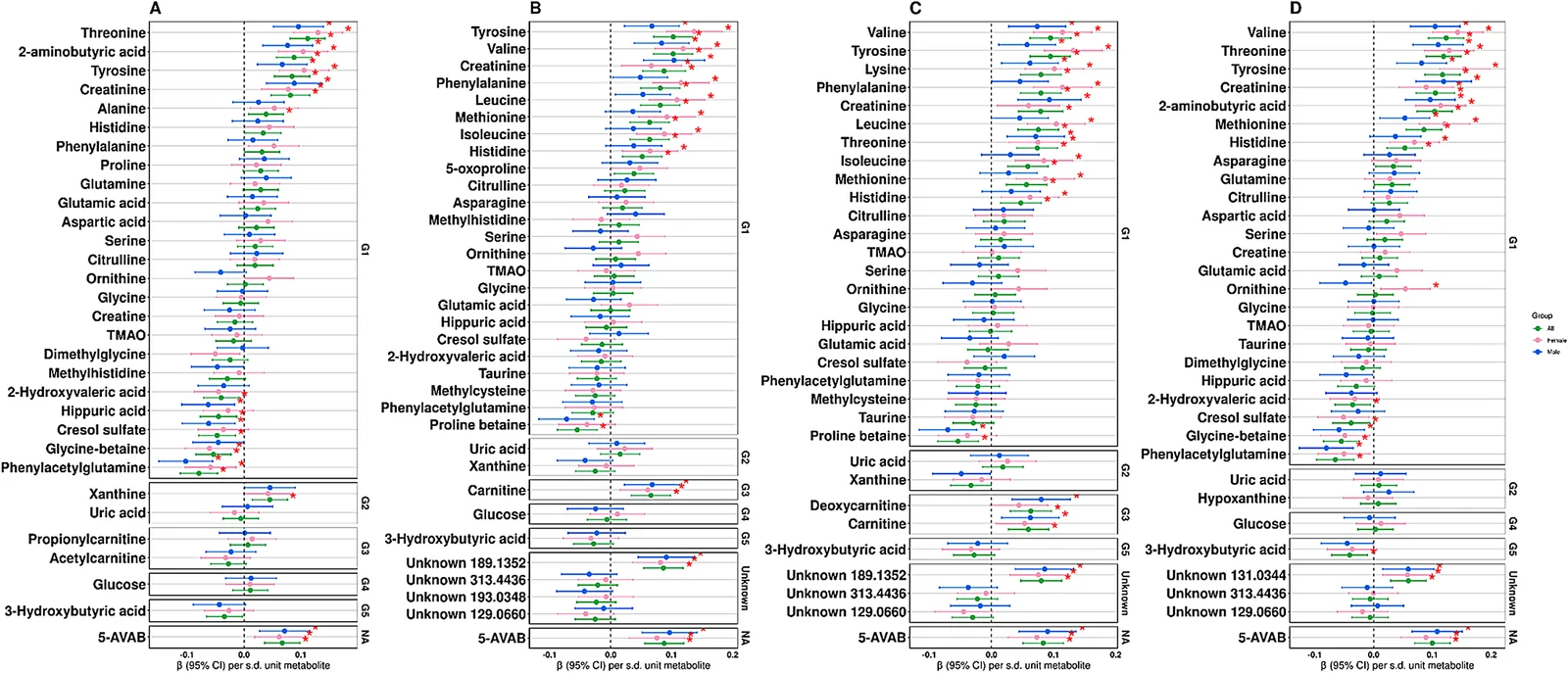
Associations between the serum metabolites and z-score child growth stratified by sex. *Notes*: Adjusted linear models were built with the following outcomes: **A**: length/height-for-age z-score. **B**: weight-for-length/height z-score. **C**: body mass index (BMI)-for-age z-score. **D**: weight-for-age z-score. The metabolites have been scaled for analysis in standard-deviation units. G1: Amino acid metabolism; G2: Purine metabolism; G3: Lipid metabolism; G4: Carbohydrate metabolism; G5: Energetic metabolism; NA: Not Available. The z-scores for anthropometric measurements were calculated according to the World Health Organization (WHO) child growth standards. Linear models were adjusted for preterm birth, per-capita family income, food diversity, food security, child development, and age. The vertical dashed line represents the absence of associations. The horizontal bars indicate the 95% confidence interval of the regression, and the midpoint of each bar represents the coefficient. Results with *p*-values < 0.05 after Benjamini–Hochberg correction for multiple comparisons were marked with a red asterisk. Green bars represent the results of the overall samples, pink bars represent the female samples, and blue bars represent the male samples. CI: confidence interval. s.d.: standard deviation. 5-AVAB: 5-aminovaleric-acid-betaine. TMAO: trimethylamine-N-oxide

After sex stratification, we found that serum glycine betaine (β = − 0.06) was inversely associated with HAZ among girls. In contrast, serum cresol sulfate (β = − 0.06) and hippuric acid (β = − 0.06) showed an inverse association with HAZ exclusively among boys (Fig. 5A). Also, serum phenylalanine (β = 0.12), leucine (β = 0.11), methionine (β = 0.09), isoleucine (β = 0.09), and histidine (β = 0.06) exhibited a direct association with WHZ, specifically among girls, whereas proline betaine (β = − 0.07) showed an inverse association exclusively among boys (Fig. 5B).

Similarly, serum phenylalanine (β = 0.12), leucine (β = 0.10), methionine (β = 0.09), isoleucine (β = 0.08), histidine (β = 0.06) showed a direct association with the BMIZ specifically among girls; however, among boys creatinine (β = 0.09), deoxycarnitine (β = 0.08) and carnitine (β = 0.06) were directly associated with the BMIZ, while proline betaine (β = − 0.07) was inversely associated (Fig. 5C).

Among girls, serum concentrations of histidine (β = 0.07) and Ornithine (β = 0.05) showed a direct association with WAZ, whereas phenylacetylglutamine (β = − 0.08) and glycine betaine (β = − 0.06) exhibited an inverse association with WAZ specifically among boys (Fig. 5D).

**Fig. 6:**
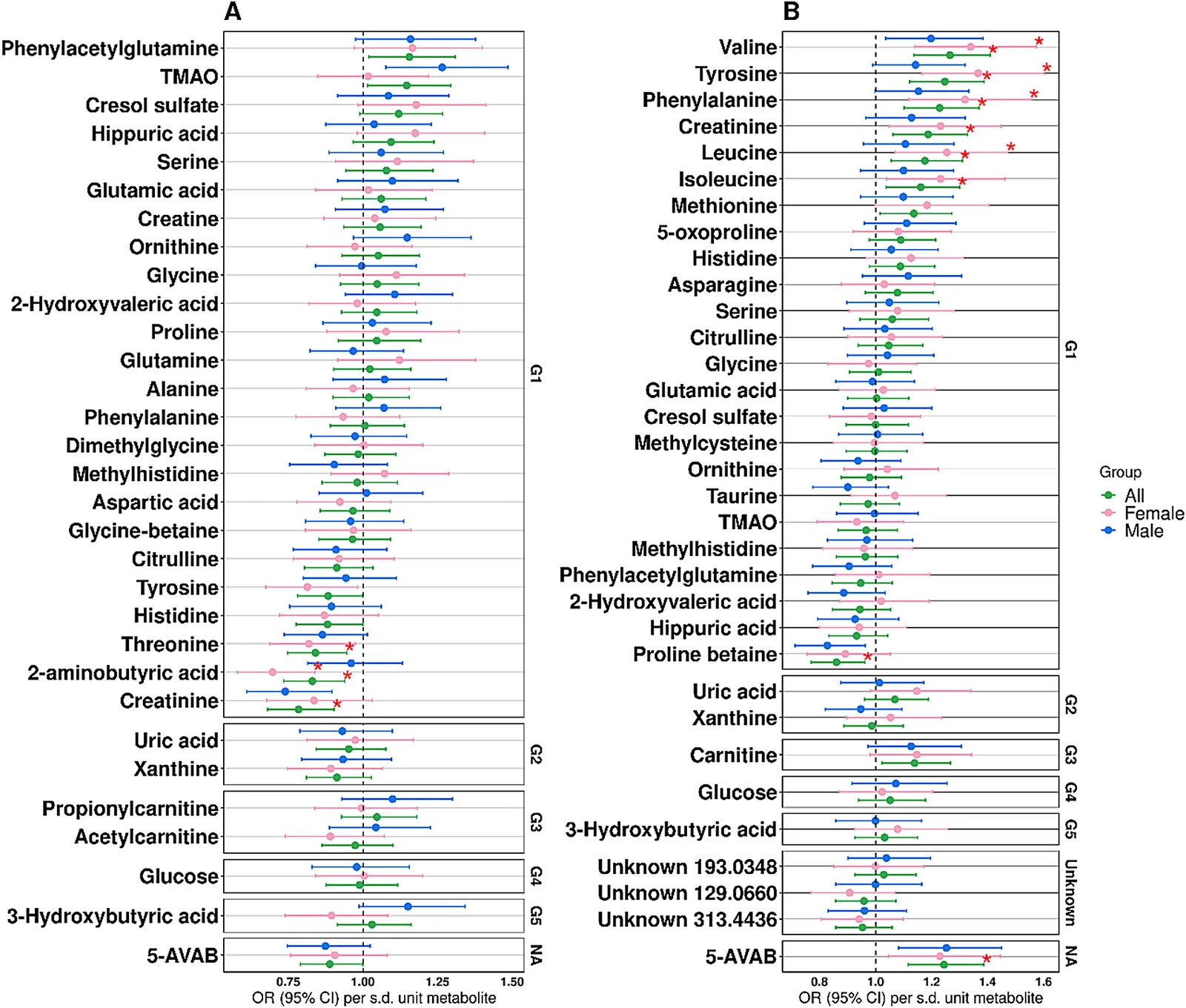
Associations between the serum metabolites and the categories of stunting and overweight stratified by sex. *Note*: Adjusted logistic models were built with the following outcomes: **A**: Stunting (length/height-for-age z-score < -2) and **B**: Overweight (weight-for-length/height z-score > 2). G1: Amino acid metabolism; G2: Purine metabolism; G3: Lipid metabolism; G4: Carbohydrate metabolism; G5: Energetic metabolism; NA: Not Available. The z-scores for anthropometric measurements were calculated according to the World Health Organization (WHO) child growth standards. The metabolites have been scaled for analysis in standard-deviation units. The logistic models were adjusted for preterm birth, per-capita family income, food diversity, food security, child development, and age. Results with *p*-values < 0.05 after Benjamini–Hochberg correction for multiple comparisons were marked with a red asterisk. Green bars represent the results of the overall samples, pink bars represent the female samples, and blue bars represent the male samples. OR: logistic regression odds ratio. CI: confidence interval, s.d.: standard deviation. 5-AVAB: 5-aminovaleric-acid-betaine. TMAO: trimethylamine-N-oxide

For stunting, only 2-aminobutyric acid showed a significant inverse association (OR = 0.70), limited to girls (Fig. 6A). We found sex-dependent differences in serum metabolites among overweight girls: tyrosine (OR = 1.37), valine (OR = 1.34), phenylalanine (OR = 1.32), and leucine (OR = 1.26) (Fig. 6B).

### Pathway analysis and network visualization

We observed metabolic pathways related to the anthropometric indicator z-score as a metabolic network. The figures show that tyrosine metabolism is related to all four anthropometric indicator z-scores, and that the metabolism of Hippurate, threonine, phenylalanine, methionine, leucine, carnitine, and valine overlaps across 2 or 3 models (Supplementary Figs. 8, 9, 10 and 11).

## Discussion

This study investigated metabolic alterations associated with early childhood malnutrition in a representative survey of children aged 5–59 months across Brazil. Our results revealed that serum creatinine is a promising anthropometric biomarker primarily associated with stunting. We also observed a direct association between branched-chain amino acids (BCAA), leucine, isoleucine, and valine, and BMIZ, WHZ, and overweight in early childhood. Overall, amino acids involved in muscle, energy, and neurotransmitter metabolism were primarily associated with excess adiposity. Sex-dependent biomarkers were also observed in our results, with a higher number of metabolites associated with amino acid metabolism in overweight girls.

Stunting is a global health issue whose prevalence is decreasing, possibly related to advances in public health policies, such as improvements in purchasing power, access to health care and education, and sanitation and hygiene [36]. In our study, stunting was significantly higher in the North region of Brazil, which has previously been reported to be associated with lower development quotient, anemia, and significant food poverty in ENANI-2019 [28, 37–41]. This shows that social inequality and limited access to adequate nutrition are associated with poor health outcomes in early childhood, with striking regional variations within countries. However, overweight/obesity remains a global problem. In the past, it was prevalent in high-income countries; nowadays, with rising consumption of ultra-processed foods due to their greater palatability and lower cost, overweight/obesity is increasing in many developing nations [5, 7, 42]. Overweight in early childhood has already been shown to affect the life course by increasing the risk of non-communicable chronic metabolic diseases [5, 43, 44].

Creatinine is a product of creatine breakdown and serves as an energy source for muscles, so low concentrations of this metabolite can be associated with loss of muscle mass [45–47]. Likewise, in the present work, this metabolite was positively associated with HAZ and negatively associated with stunting, indicating that short children have lower creatinine concentrations [46]. This metabolite was also positively associated with WHZ, BMIZ, and WAZ; in the sex-stratified analysis for BMIZ, we found an association only in boys, which aligns with other studies showing the sex-dependent nature of this metabolite [42, 48, 49]. These results provide insight into the tracking of creatinine in public health to identify risks to child growth.

5-AVAB is a microbially derived metabolite that has previously been identified as a dietary biomarker of whole grain intake and decreases the β-oxidation of fatty acids and inhibits the biosynthesis of *L*-carnitine [50, 51]. Recently, 5-AVAB has been associated with an obesogenic dietary pattern linked to whole-body fat mass and muscle insulin resistance, but negatively associated with lean body mass [50, 51]. A recent weight management intervention in overweight and obese individuals reported that higher serum 5-AVAB levels were associated with worse glucose metabolism and hepatic steatosis [51]. Most studies to date have implicated a deleterious effect of 5-AVAB on the brain-gut-liver metabolic axis, consistent with our findings of a robust positive association with WHZ, BMIZ, WAZ, and overweight in early childhood; however, we have also found a direct association with HAZ, which would not be expected.

We identified three metabolites that are interconnected within the same metabolic pathway: tyrosine, phenylalanine, and cresol sulfate. In our results, tyrosine was positively associated with all child growth z-scores and overweight. Phenylalanine also presented a positive association with WHZ, BMIZ, and overweight, while cresol sulfate demonstrated a negative association with HAZ and WAZ. Phenylalanine is a precursor of tyrosine, which acts in catecholamine synthesis. Tyrosine must be rapidly metabolized, as its accumulation may be related to vitamin C deficiency, which could suggest a lower intake of fruits and phenolic compounds in overweight children, although dietary data were not used to test this hypothesis [46, 52, 53]. Cresol sulfate is a uremic toxin and a gut flora metabolite that is a product of the fermentation of tyrosine [46, 54]. In our previous work, cresol sulfate was negatively associated with the child development quotient, and this compound is often elevated in patients with chronic kidney disease. It has also been associated with cardiovascular and oxidative injury [15, 54, 55].

An interesting finding was a positive association between BCAA and WHZ, BMIZ, and overweight, and between valine and WAZ. They are essential amino acids involved in stress, energy, and muscle metabolism, with each directed toward its respective pathway function: valine goes through carbohydrates (glycogenic), leucine to lipids (ketogenic), and isoleucine goes through both glycogenic and ketogenic pathways. Consistent with our findings, the metabolites leucine and valine have previously been positively associated with BMIZ [56, 57]. However, valine deficiency has previously been associated with abnormalities in fine motor coordination, underscoring the importance of maintaining a balanced level of this metabolite in the organism [58].

Additionally, the metabolites phenylalanine and the BCAAs were positively associated only with girls in the sex-stratified analysis, which aligns with findings from Azab et al. [11], who used data from two Canadian cohort studies of children aged 5 years [11]. Sex dependence in adiposity is currently being studied; it may be mediated by sex hormones, related to differences in maturation time and mitochondrial adaptation, and could reflect anatomical and functional disparities between males and females [59, 60]. A systematic review and meta-analysis have found that leptin levels were higher in girls, a condition commonly associated with obesity [61].

We observed that the essential amino acid threonine was positively associated with HAZ, BMIZ, and WAZ, and inversely associated with the stunting category. This compound is involved in protein synthesis and is found in the human diet, including poultry, fish, meat, lentils, black beans, and sesame seeds [62]. An interesting point is that it is found in higher amounts in infant formula than in human breast milk, which can result in the accumulation of this amino acid in the brain and affect brain development; nonetheless, adequate threonine in breast milk is considered a protective factor for the gut microbiome and the immune system [63, 64]. Our findings on BMIZ and WAZ are also consistent with those of Rafiq et al. [65], who found that threonine was positively associated with overweight/obesity, underscoring the need to maintain balance within the organism [65].

Another important metabolite associated with WHZ and BMIZ was carnitine, a quaternary ammonium compound synthesized from lysine and methionine that plays a role in lipid metabolism during the beta-oxidation of fatty acids [46]. Our findings align with the literature, in which circulating carnitine has been positively associated with overweight and obesity in children [56, 65, 66]. We also hypothesize that this result may be related to infant formula consumption, because in another study, carnitine was directly associated with low birth weight, and most of these children were fed formula [66].

In contrast to most of our results, in which the described serum metabolites showed positive associations with WHZ, BMIZ, and overweight, proline betaine demonstrated a negative association, indicating a protective effect against excess adiposity. This result could be explained by proline betaine, an exogenous dietary betaine that may play roles in signaling and have anti-inflammatory effects and is recognized as a biomarker of citrus fruit consumption [56, 67, 68]. We hypothesize that children with low citrus fruit intake may have greater adiposity, which may also reflect a poor diet quality. Further studies are needed to validate these findings.

This study has several strengths. It represents a pioneering effort in Brazil, i.e., the first population-based survey of 6-59-month-old children, with the serum metabolic phenotype characterized for 4978 participants randomly and representatively selected from the total collected sample. MSI-CE-MS provided a high-throughput, low-cost platform for metabolomic analyses that is optimal for large-scale epidemiological studies and exhibits excellent long-term data fidelity [16, 20]. We used robust statistical analyses, DAGs to minimize bias risk, and BH correction to control false positives from multiple testing. However, we acknowledge some limitations. Metabolome coverage was limited to 73 polar/ionic metabolites because the small on-column injection volume reduced concentration sensitivity; water-insoluble lipids were not analyzed because they require non-aqueous buffer systems [69]. Although the 4978 serum samples were randomly selected to reproduce the distribution of the enrolled children, selection bias cannot be entirely ruled out, since children with a blood specimen may differ from those without one. Other limitations include the cross-sectional design, which precludes causal inference; the small number of cases in the categorical analyses; and the absence of longitudinal data.

## Conclusion

In conclusion, our findings suggest differences in the metabolic signature across standard growth conditions and that this signature shows some sex dependence. These results provide novel insights into the biological pathways underlying malnutrition and may inform future strategies for early identification and prevention. The present study is exploratory, and prospective longitudinal studies would be valuable for comparison with our findings. To our knowledge, this is the largest-sample-size study using a serum metabolomics approach in early childhood, and these results will contribute to the current literature by addressing existing gaps.

## Supplementary Information

Below is the link to the electronic supplementary material.

Supplementary Material 1

## Data Availability

Data described in the manuscript, codebook, and analytic code will be made available upon request, subject to the corresponding author.

## Abbreviations

5-AVAB: 5-aminovaleric acid betaine or δ-valerobetaine
BCAA: Branched-chain amino acids
BMIZ: Body mass index for age z-score
CE: Capillary electrophoresis
CV: Coefficient of variation
DAG: Directed acyclic graph
DQ: Developmental quotient
EBIA: Brazilian household food insecurity measurement scale
ECD: Early childhood development
ENANI-2019: Brazilian National Survey on Child Nutrition
HAZ: Height-for-age z-score
JME: Joint child malnutrition estimates
MDD: Minimum dietary diversity
MSI-CE-MS: Multisegment injection-capillary electrophoresis-mass spectrometry
PLS-R: Partial least squares regression
QC: Quality control
QRILC: Quantile regression imputation of left-censored data
RMT: Relative migration time
RPA: Relative peak areas
SWYC: Survey of well-being of young children
TMAO: Trimethylamine-N-oxide
VIP: Variable importance in projection
WAZ: Weight-for-age z-score
WHO: World health organization
WHZ: Weight-for-height z-score
