# Supplementary material for: Serum metabolic signatures of nutritional status in early childhood: results from the Brazilian National Survey on Child Nutrition (ENANI-2019)
Source: Eur J Nutr. 2026 Sep 12;65(7):252. doi: 10.1007/s00394-026-04101-9 (PMC13570930; doi:10.1007/s00394-026-04101-9)
Supplement: Supplementary file 1 [file 394_2026_4101_MOESM1_ESM.docx]

**Supplementary Table 1** - Association between metabolites and sex.

| **Metabolites** | **p-value** | **coefficients** | **Lower_CI** | **Upper_CI** | **Significant** |
| --- | --- | --- | --- | --- | --- |
| Ornithine | 4.68 x 10 ^ (-5) | 0.05 | 0.03 | 0.07 | * |
| Creatinine | 6.61 x 10 ^ (-5) | -0.04 | -0.06 | -0.02 | * |
| Oxoproline | 4.28 x 10 ^ (-4) | 0.05 | 0.02 | 0.08 | * |
| Lysine | 6.11 x 10 ^ (-4) | 0.04 | 0.02 | 0.06 | * |
| 2-aminobutyric acid | 1.41 x 10 ^ (-3) | 0.04 | 0.01 | 0.06 | * |
| Homovanillic acid | 0.01 | -0.02 | -0.04 | -0.01 | * |
| Unknown 189.1352 | 0.02 | 0.03 | 0.00 | 0.05 | * |
| Glycine betaine | 0.02 | 0.02 | 0.00 | 0.04 | * |
| Propionylcarnitine | 0.02 | -0.03 | -0.06 | 0.00 | * |
| 5-AVAB | 0.04 | -0.03 | -0.07 | 0.00 | * |
| Unknown 129.0660 | 0.04 | -0.02 | -0.04 | 0.00 | * |
| Glucose | 0.04 | -0.06 | -0.11 | 0.00 | * |
| Tyrosine | 0.05 | -0.02 | -0.04 | 0.00 | * |
| Deoxycarnitine | 0.08 | -0.02 | -0.04 | 0.00 |  |
| Tryptophan | 0.09 | -0.03 | -0.06 | 0.00 |  |
| Choline | 0.13 | 0.04 | -0.01 | 0.09 |  |
| Creatine | 0.14 | -0.01 | -0.03 | 0.00 |  |
| TMAO | 0.16 | -0.03 | -0.07 | 0.01 |  |
| Glutamine | 0.18 | 0.04 | -0.02 | 0.11 |  |
| Unknown 135.0293 | 0.20 | -0.01 | -0.04 | 0.01 |  |
| Arginine | 0.21 | -0.03 | -0.09 | 0.02 |  |
| Xanthine | 0.23 | 0.02 | -0.01 | 0.04 |  |
| Hippuric acid | 0.23 | -0.03 | -0.07 | 0.02 |  |
| Cresol sulfate | 0.23 | 0.03 | -0.02 | 0.07 |  |
| 2-Hydroxybutyric acid | 0.24 | 0.01 | -0.01 | 0.04 |  |
| Unknown 276.1191 | 0.25 | 0.03 | -0.02 | 0.07 |  |
| Histidine | 0.25 | 0.01 | -0.01 | 0.02 |  |
| Alanine | 0.26 | 0.01 | -0.01 | 0.04 |  |
| Carnitine | 0.26 | -0.01 | -0.03 | 0.01 |  |
| Betaine | 0.27 | 0.02 | -0.01 | 0.05 |  |
| 3-Hydroxybutyric acid | 0.28 | 0.02 | -0.02 | 0.06 |  |
| Methylcysteine | 0.33 | 0.02 | -0.02 | 0.05 |  |
| Threonine | 0.33 | 0.01 | -0.01 | 0.03 |  |
| Leucine | 0.34 | -0.01 | -0.04 | 0.01 |  |
| Isoleucine | 0.36 | -0.01 | -0.04 | 0.01 |  |
| Unknown 193.0348 | 0.39 | -0.01 | -0.04 | 0.01 |  |
| ADMA | 0.40 | -0.01 | -0.03 | 0.01 |  |
| Unknown 161.1281 | 0.40 | -0.02 | -0.05 | 0.02 |  |
| Glutamic acid | 0.45 | 0.01 | -0.02 | 0.05 |  |
| Phenylalanine | 0.45 | -0.01 | -0.03 | 0.01 |  |
| Cysteine-S-sulfate | 0.45 | -0.01 | -0.03 | 0.01 |  |
| Asparagine | 0.48 | -0.01 | -0.05 | 0.03 |  |
| alpha-Ketoisovaleric acid | 0.50 | -0.01 | -0.03 | 0.02 |  |
| Citrulline | 0.52 | -0.01 | -0.03 | 0.01 |  |
| Acetylcarnitine | 0.52 | -0.01 | -0.04 | 0.02 |  |
| Unknown 131.0344 | 0.52 | -0.01 | -0.04 | 0.02 |  |
| Unknown 334.6885 | 0.53 | 0.02 | -0.03 | 0.06 |  |
| Serine | 0.56 | 0.02 | -0.04 | 0.07 |  |
| Methionine | 0.61 | 0.01 | -0.02 | 0.03 |  |
| Cystine | 0.64 | -0.01 | -0.05 | 0.03 |  |
| Methylhistidine | 0.65 | -0.01 | -0.05 | 0.03 |  |
| Aspartic acid | 0.65 | 0.01 | -0.03 | 0.04 |  |
| Lactic acid | 0.67 | 0.01 | -0.02 | 0.04 |  |
| Proline | 0.67 | 0.01 | -0.03 | 0.05 |  |
| Hydroxyproline | 0.68 | 0.00 | -0.02 | 0.03 |  |
| Hypoxanthine | 0.68 | 0.01 | -0.04 | 0.06 |  |
| Taurine | 0.70 | 0.00 | -0.02 | 0.03 |  |
| Unknown 313.4436 | 0.75 | -0.01 | -0.04 | 0.03 |  |
| Pyruvic acid | 0.76 | 0.01 | -0.04 | 0.06 |  |
| Cysteinylglycine disulfide | 0.77 | 0.00 | -0.03 | 0.02 |  |
| Dimethylglycine | 0.77 | 0.00 | -0.02 | 0.02 |  |
| F-Phe Neg | 0.77 | 0.00 | 0.00 | 0.01 |  |
| Valine | 0.79 | 0.00 | -0.02 | 0.02 |  |
| 2-Hydroxyvaleric acid | 0.80 | 0.00 | -0.03 | 0.02 |  |
| Unknown 471.7380 | 0.80 | -0.01 | -0.05 | 0.04 |  |
| Unknown 209.0297 | 0.83 | 0.00 | -0.02 | 0.02 |  |
| Unknown 117.0552 | 0.84 | 0.00 | -0.02 | 0.03 |  |
| Inorganic phosphate | 0.84 | 0.00 | -0.02 | 0.02 |  |
| Uric acid | 0.86 | 0.00 | -0.02 | 0.02 |  |
| Proline betaine | 0.90 | 0.01 | -0.08 | 0.09 |  |
| F-Phe Pos | 0.91 | 0.00 | 0.00 | 0.00 |  |
| 3-Methyl-2-oxovaleric acid | 0.93 | 0.00 | -0.03 | 0.03 |  |
| Phenylacetylglutamine | 0.96 | 0.00 | -0.04 | 0.04 |  |

**Notes**: The metabolites have been scaled to be analyzed in standard deviation units. The sex categories were defined as male = 0 and female = 1; P-values were adjusted for multiple comparisons using the Benjamini–Hochberg correction. 5-AVAB = 5-aminovaleric-acid-betaine. TMAO = trimethylamine-N-oxide

**Supplementary Table 2 -** Association between stunting and overweight malnutrition with per capita family income and macro-region.

|  | **With stunting (%)** | **Without stunting (%)** | **p-value** | **With overweight (%)** | **Without overweight (%)** | **p-value** |
| --- | --- | --- | --- | --- | --- | --- |
| **Macroregion** |  |  |  |  |  |  |
| Midwest | 1.2 | 19.4 | 0.01 | 1.1 | 19.5 | 0.0001 |
| North | **1.9** | **23.0** |  | 1.5 | 23.4 |  |
| Northeast | 0.9 | 21.6 |  | 1.7 | 20.8 |  |
| South | 0.7 | 12.8 |  | **1.5** | **12.1** |  |
| Southeast | 1.0 | 17.5 |  | 1.5 | 16.9 |  |
| **Per-capita family income (USD)‡** | |  |  |  |  |  |
| < 62.20 | 2.2 | 29.2 | 0.04 | 1.9 | 29.5 | 0.12 |
| 62.20-124.40 | 1.9 | 32.0 |  | 2.6 | 31.4 |  |
| 124.50-248.70 | 1.2 | 23.3 |  | 2.0 | 22.5 |  |
| >248.70 | 0.4 | 9.7 |  | 0.8 | 9.3 |  |
| **Total (%)** | 5.7 | 94.3 | - | 7.3 | 92.7 | - |
| **Notes**: Values in bold indicate statistical significance based on the cellwise adjusted residual using the adjusted alpha level (MacDonald *et al*., 2000). | | | | | | |

**Supplementary Figure 1** - Directed acyclic graph (DAG) of the associations between child serum metabolome and child nutritional status. **Notes**: Breastfeeding refers to breastfeeding practice exclusively until 6 months and/or complemented until 2 years. Minimum adjustments suggested by the DAG inlcude birth weight, preterm birth, per capita family income, food diversity, food security, child development, sex, age, and breastfeeding. Blue rectangles: ancestor of the outcome; pink rectangles: ancestor of exposure and outcome; white circles: non-observed variables in the ENANI-2019.

**Supplementary Figure 2 –** Flowchart of the study analytical pipeline. **Notes**: ¹Multi-Segment Injection-Capillary Electrophoresis-Mass Spectrometry; ² World Health Organization.

**Supplementary Figure 3 -** Identification of an unknown cation (160.132 *m/z*) in serum that was consistently associated with all four anthropometric measures in the ENANI-2019 study (together with creatinine and tyrosine) when using collisional-induced dissociation experiments (10 V) by CE-MS/MS in positive ion mode. The characteristic neutral loss of trimethylamine (59.072 *m/z*) and formation of a product ion as a base peak (101.060 *m/z*) is consistent with 5-aminovaleric acid betaine (5-AVAB) or d-valerobetaine.

**Supplementary Figure 4** - Residual means squared error (RMSEP) and R squared across Partial Least Square Regression (PLSR) models with different numbers of components to length/height for age z-score outcome. **Note**: Training and test datasets were done with 80% (n = 4003) and 20% (n = 1001) of observations, respectively.

**Supplementary Figure 5** - Residual means squared error (RMSEP) and R squared across Partial Least Square Regression (PLSR) models with different numbers of components to weight for length/height z-score outcome. **Note**: Training and test datasets were done with 80% (n = 4003) and 20% (n = 1001) of observations, respectively.

**Supplementary Figure 6** - Residual mean squared error (RMSEP) and R-squared across Partial Least Squares Regression (PLSR) models with different numbers of components to body mass index (BMI) for age z-score outcome. **Note**: Training and test datasets were done with 80% (n = 4003) and 20% (n = 1001) of observations, respectively.

**Supplementary Figure 7** - Residual mean squared error (RMSEP) and R-squared across Partial Least Squares Regression (PLSR) models with different numbers of components to weight for age z-score outcome. **Note**: Training and test datasets were done with 80% (n = 4003) and 20% (n = 1001) of observations, respectively.

**Supplementary Figure 8** - Metabolic network based on metabolites differentially associated with length/height-for-age z-score. **Notes**: Each node represents a metabolite, and the lines indicate biochemical connections among metabolic pathways.

**Supplementary Figure 9** - Metabolic network based on metabolites differentially associated with weight-for-length/height z-score. **Notes**: Each node represents a metabolite, and the lines indicate biochemical connections among metabolic pathways.

**Supplementary Figure 10** - Metabolic network based on metabolites differentially associated with body mass index (BMI)-for-age z-score. **Notes**: Each node represents a metabolite, and the lines indicate biochemical connections among metabolic pathways..

**Supplementary Figure 11**- Metabolic network based on metabolites differentially associated with weight-for-age z-score. **Notes**: Each node represents a metabolite, and the lines indicate biochemical connections among metabolic pathways.
